# Effect of blood groups on acquired and congenital thrombotic thrombocytopenic purpura and clinical correlation: Multi-center Turkish cohort study

**DOI:** 10.1016/j.htct.2024.09.2483

**Published:** 2025-02-19

**Authors:** Cevat İlteriş Kıkılı, Damla Ortaboz, Melek Yanaşık, Muhlis Cem Ar, Sevgi Kalayoğlu Beşışık

**Affiliations:** Istanbul University, Istanbul Faculty of Medicine, Department of Internal Medicine, Istanbul, Turkey

**Keywords:** TTP, Abo blood group, Thrombotic thrombocytopenic purpura, TMA, Thrombotic microangiopathy

## Abstract

**Background:**

Thrombotic thrombocytopenic purpura (TTP) is a microangiopathic hemolytic anemia associated with ADAMTS-13 deficiency, a cleaving protease of von Willebrand factor (vWF). According to the literature, blood group O tends to be less common among these patients than in the general population. This study aimed to investigate whether the decreasing trend of blood group O in thrombotic thrombocytopenic purpura patients is observed in a Turkish cohort and to analyze the relationship between clinical outcomes and blood groups.

**Patients and Methods:**

A total of 65 patients with acquired and five patients with congenital thrombotic thrombocytopenic purpura from two university hospitals were enrolled in this study. As a control group, the blood group data of 136,231 individuals who were not diagnosed without anemia were obtained from the archives of the Istanbul Medical Faculty Blood Centre. The blood groups were compared between cases and the Control Group using the chi-square test. Subsequently, the clinical outcomes of patients and categorized blood groups were compared by the chi-square test, Mann Whitney U test and Cox regression with Kaplan Meier analysis.

**Results:**

This study shows that the decreasing trend of blood group O was not observed in this Turkish cohort. Regarding the relationship between blood groups and clinical outcomes, the AB blood group is associated with a good prognosis and blood group O is associated with a poor prognosis. In addition, relapses were more common with blood group A patients but less common in blood group B.

**Conclusion:**

The current study shows the association between thrombotic thrombocytopenic purpura and blood groups in the Turkish cohort. This study also contributes by analyzing the relationship between blood groups and clinical outcomes.

## Introduction

Thrombotic Thrombocytopenic Purpura (TTP) is an extremely rare microangiopathic hemolytic anemia characterized by a deficiency of ADAMTS-13, a von Willebrand Factor (vWF) cleaving protease. ADAMTS-13 deficiency leads to ultra-large vWF multimers. The interaction of these multimers with platelets results in thrombus formation in microvascular vessels leading to organ ischemia. If not treated properly and promptly, it may be fatal.^1^, ^2^, ^3^, ^4^, ^5^

Congenital TTP is the type of TTP caused by a mutation of the gene encoding ADAMTS-13 and accounts for approximately 10 % of TTP patients with >150 mutations of the gene encoding ADAMTS 13 having been identified so far. The autoimmune type of TTP, which causes ADAMTS-13 deficiency as a result of the formation of antibodies against ADAMTS-13, is known as acquired TTP and accounts for approximately 90 % of TTP.^1^, ^2^, ^3^, ^4^, ^5^^,^^6^^,^^7^

It has previously been reported that blood group antigens have different sensitivities to vWF. Accordingly, blood group A and B antigens were found to be protective against vWF. The cleavage of vWF by ADAMTS-13 has been found to be faster in blood group O individuals and slower in blood group AB. According to the literature, blood groups are responsible for up to 40 % of the variation in vWF levels in plasma: thus, vWF levels range in the decreasing order blood group AB>*B* > *A* > *O*>Bombay. Therefore, according to the literature, blood group O tends to be less common among TTP patients than in the general population.^8^, ^9^, ^10^

### Objective

This study aimed to determine whether the decreasing trend of blood group O among TTP patients is observed in a Turkish cohort and to determine the relationship between the clinical outcomes of TTP and blood groups.

## Methods

### Patients

This study enrolled 65 acquired and five congenital TTP patients who were diagnosed based on clinical and laboratory data and followed up at the Department of Hematology, Istanbul Faculty of Medicine, Istanbul University and Department of Hematology, Cerrahpaşa Faculty of Medicine, Istanbul University Cerrahpaşa. Verbal and written informed consent was obtained from participants. This study was conducted in accordance with the Declaration of Helsinki with the approval of the Istanbul Medical Faculty Clinical Research Ethics Committee.

The control group comprised the blood group data of 136,231 patients without TTP, who were followed up between 1 January 2014 and 1 January 2020 in the Istanbul Faculty of Medicine Blood Center.

## Methods

The blood groups of the patients and Control Group were categorized as A, B, O, AB, and Rh(D) antigen and the groups were compared using the chi-square test. Comparisons between congenital TTP patients and the Control Group could not be performed due to the small number of patients.

Subsequently, patients were categorized separately according to the presence or absence of thrombosis, renal involvement, fever, neurological involvement and relapse. The categorized data were compared separately between blood groups using the chi-square test. Consequently, logistic regression analyses were performed and odds ratios (ORs) were calculated with 95 % confidence intervals (95 % CI).

Hemoglobin, platelet, lactate dehydrogenase (LDH), indirect bilirubin, creatinine and ADAMTS-13 inhibitor levels of the patients were tested for normality during crises, using the Q-Q plot, histogram, skewness-kurtosis and Kolmogorov-Smirnov methods. Since the data were not normally distributed, the data were compared separately between blood groups using the Mann-Whitney U test. The data are presented as medians and interquartile range.

Furthermore, LDH and platelet normalization (platelet count >150×10^9^/L) from the time of crises were compared separately between blood groups using COX regression analysis. Hazard ratios were calculated with 95 % CIs. Kaplan-Meier analysis was performed using the log-rank method for significant values and presented graphically.

Statistical analyses were performed using the Statistical Package for Social Sciences (SPSS - IBM version 26.0). *P*-values <0.05 was considered statistically significant.

## Results

The blood groups of 136,231 healthy controls and 65 acquired and five congenital TTP patients were categorized as A, B, O, AB, and Rh(D) antigen and their frequencies and percentages were determined (Table 1).

**Table 1:** Frequency and percentage of blood groups of congenital and acquired TTP patients and the Control Group.

Blood group	Congenital TTP n (%)	Acquired TTP n (%)	Control Group n (%)	*p*-value (chi-square)
**A**	2 (40)	28 (43.1)	57,059 (41.89)	0.845
**B**	0 (0.0)	9 (13.8)	20,790 (15.26)	0.751
**AB**	0 (0.0)	7 (10.8)	10,806 (7.93)	0.397
**O**	3 (60)	21 (32.3)	47,576 (34.93)	0.658
**Rh(D)**	5 (100)	56 (86.2)	115,819 (85.02)	0.797

**Figure 1:**
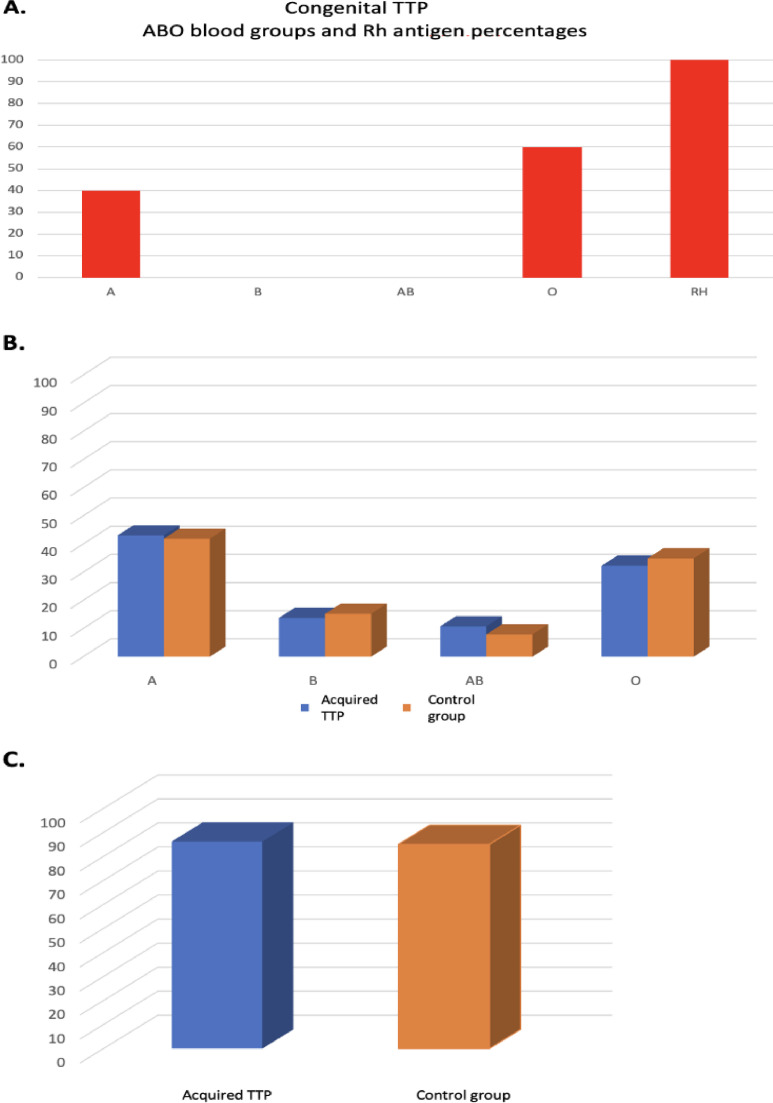
A congenital TTP ABO blood group and Rhantigen percentages. (B) Comparison of the percentages of ABO blood groups in acquired TTP and control group. (C) Comparison of the percentages of Rh antigen in acquired TTP and control group.

The results in Table 1 are presented graphically in Figure 1A, 1B and 1C.

Subsequently, clinical parameters of TTP patients were compared separately between blood groups.

### Thrombosis

All sizes and types of blood clots in any organ at the time of crisis were included.

Thrombosis was detected in 22.5 % (*n* = 9/40) of non-A blood group patients and 36.7 % (*n* = 11/30) of A blood group patients (*p*-value = 0.194; OR: 1.994; 95 % CI: 0.698–5.698).

Thrombosis was detected in 30.2 % (*n* = 19/63) of non-AB blood group patients and 14.3 % (*n* = 1/7) of AB blood group patients (*p*-value = 0.378; OR: 0.386; 95 % CI: 0.043–3.429).

Thrombosis was detected in 31.1 % (*n* = 19/61) of non-B blood group patients and 11.1 % (*n* = 1/9) of B blood group patients (*p*-value = 0.214; OR: 0.276; 95 % CI: 0.032–2.368).

The Rh and O blood groups were not found to have any significant effect on the risk of thrombosis.

### Renal involvement

Renal involvement was detected in 34.9 % (*n* = 22/63) of non-AB blood group patients and 0.0 % of AB blood group patients (*p*-value = 0.059) The OR could not be calculated since renal involvement was not observed in the AB blood group.

The Rh, A, B, and O blood groups were not found to have any significant effect on the risk of renal involvement.

### Neurological involvement

Neurological involvement was detected in 68.3 % (*n* = 41/60) of non-B blood group patients and 88.9 % (*n* = 8/9) of B blood group patients (*p*-value = 0.205; OR: 3.707; 95 % CI: 0.432–31.790).

The RH, A, O, and AB blood groups were not found to have any significant effect on the risk of neurological involvement.

### Fever

Fever was detected in 11.1 % (*n* = 1/9) of Rh-negative patients and 41.0 % (*n* = 25/61) of Rh-positive patients (*p*-value = 0.083; OR: 5.556; 95 % CI: 0.653–47.246).

Fever was detected in 34.9 % (*n* = 22/63) of non-AB blood group patients and 57.1 % (*n* = 4/7) of AB blood group patients (*p*-value = 0.248; OR: 2.485; 95 % CI: 0.510–12.113).

The O, A and B blood groups were not found to have any significant effect on the risk of fever.

### Relapse

Relapse was detected in 28.9 % (*n* = 11/38) of non-A blood group patients and 60 % (*n* = 18/30) of A blood group patients (*p*-value = 0.010; OR: 3.682; 95 % CI: 1.338–10.134). The A blood group was found to be a statistically significant risk factor for relapse.

Relapse was detected in 47.5 % (*n* = 28/59) of non-B blood group patients and 11.1 % (*n* = 1/9) of B blood group patients (*p*-value = 0.040; OR: 0.138; 95 % CI: 0.016–1.177). The B blood group was found to be a statistically significant protective factor for relapse.

The Rh, AB and O blood groups were not found to have any significant effect on the risk of relapse.

### Hemoglobin nadir at the time of crises

The A, B, O, AB and Rh blood groups were analyzed separately and there was no significant effect on the hemoglobin nadir in TTP patients at the time of the crisis.

### Platelet count nadir at the time of crises

The mean platelet count nadir at the time of the crisis was 11.75×10^9^/L (range: 7.00–19.75×10^9^/L) in 36 patients in the non-A blood groups, whereas the mean platelet count nadir at the time of the crisis was 19.20×10^9^/L (range: 10.00–34.60×10^9^/L) in 26 patients with the A blood group (*p*-value = 0.007). The association between blood group A and the platelet count nadir at the time of the crisis is shown in Table 2A.

**Table 2A:** The association between blood group A and the platelet count nadir at the time of the crisis.

Platelet count	A blood group (*n* = 36)	Non-A blood groups (*n* = 26)
median (IQR) × 10^9^/L	11.75 (7.00–19.75)	19.20 (10.00–34.60)
*p*-value = 0.007^m^		

IQR: Interquartile range.

m = Mann-Whitney U test. *p*-value <0.05 are statistically significant.

The mean platelet count nadir at the time of the crisis was 15.25×10^9^/L (range: 9.00–32.00×10^9^/L) in 42 patients with non-O blood groups, whereas the mean platelet count nadir at the time of the crisis was 9.80×10^9^/L (range: 7.00–19.75×10^9^/L) in 20 patients with the O blood group (*p*-value = 0.077). The association between blood group O and the platelet count nadir at the time of the crisis is shown in Table 2B.

**Table 2B:** The association between blood group O and the platelet count nadir at the time of the crisis.

Platelet count	O blood group (*n* = 42)	Non-O blood groups (*n* = 20)
median (IQR) x 10^9^/L	15.25 (9.00–32.00)	9.80 (7.00–19.75)
*p*-value = 0.077^m^		

IQR: Interquartile range.

m = Mann-Whitney U test. *p*-value <0.05 are statistically significant.

The B, AB and Rh blood groups were analyzed separately and there were no significant effects on the platelet count nadir in TTP patients at the time of crises.

### Peak LDH level at the time of crises

A, B, O, AB, and Rh blood groups were analyzed separately and there were no significant effects on the peak LDH level in TTP patients at the time of crises.

### Indirect bilirubin at the time of crises

A, B, O, AB and Rh blood groups were analyzed separately and there were no significant effects on the indirect bilirubin level in TTP patients at the time of crises.

### Creatinine at the time of crises

A, B, O and Rh blood groups were analyzed separately and there were no significant effects on the creatinine level in TTP patients at the time of crises.

In the AB blood group, the creatinine level at the time of the crisis was 0.94 mg/dL (range: 0.71–1.46 mg/dL) in 46 patients with non-AB blood groups and 0.67 mg/dL (range: 0.59–0.80 mg/dL) in six AB blood group patients (*p*-value = 0.016 - Table 3).

**Table 3:** The association between the AB blood group and creatinine level at the time of crises.

Creatinine	AB blood group (*n* = 46)	Non-AB blood group (*n* = 6)
median (IQR) – mg/dL	0.94 (0.71–1.46)	0.67 (0.59–0.80)
*p*-value = 0.016^m^		

IQR: Interquartile range.

m = Mann-Whitney U test. *p*-value <0.05 are statistically significant.

### ADAMTS-13 inhibitor level at the time of crises

A, B, O, AB and Rh blood groups were analyzed separately and there were no significant effects on ADAMTS-13 inhibitor levels in patients with acquired TTP at the time of crises.

### LDH normalization after crises

A, B, O, AB and Rh blood groups were analyzed separately and there were no significant effects on the LDH normalization time in TTP patients after crises.

### Platelet normalization after crises

A, B, O and Rh blood groups were analyzed separately and there were no significant effects on the platelet normalization time in TTP patients after crises.

**Figure 2:**
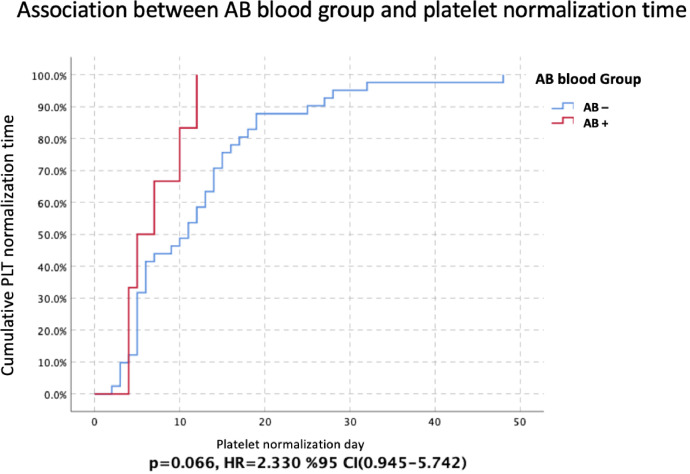
The association between the AB blood group and platelet normalization after crises (days) (Kaplan-Meier analysis with the log-rank method). 95 % CI: 95 % confidence intervals; HR: Hazard ratio; PLT: Platelet count; AB-: Non-AB blood groups; AB+: AB blood group *p* < 0.05 was considered statistically significant.

The mean platelet normalization time was 7.0 ± 1.366 (95 % CI: 4.322–9.678) in patients with AB blood group, whereas the mean platelet normalization time was 12.146 ± 1.468 (95 % CI: 9.269–15.024) in patients without the AB blood group (*p*-value = 0.066 - Cox regression analysis; Hazard ratio: 2.330; 95 % CI: 0.945–5.742 - Figure 2).

## Discussion

In the literature, a study conducted by Zuberi et al. on 74 idiopathic TTP patients evaluated the incidence, mortality and recurrence for each blood group separately. Accordingly, the O blood group was 36 %, the A blood group 36 %, the B blood group 25 % and the AB blood group 2 % of TTP patients. In the Detroit area population, the results were 44 %, 33 %, 20 % and 3 % for the O, A, B and AB blood groups, respectively. Thus, the O blood group was found to have a decreasing trend in TTP patients however, no statistically significant difference was found and no difference was found between blood groups in terms of relapse and deaths.^8^

In the study conducted by Staropoli et al., the clinical characteristics of 76 sporadic TTP patients were analyzed according to blood groups. There was no difference in clinical parameters between patients with the O blood group and those of non-O blood groups.^9^

In another study conducted by Hussein et al., the blood group distribution of 33 TTP patients was analyzed and it was determined that the O blood group was found to be 12 %, that is, less than expected (30 %). In addition, the number of plasmapheresis, recurrence rates and mortality rates were compared between blood groups. Consequently, it was observed that more plasmapheresis sessions were required to achieve remission in the O blood group compared to the B blood group (*p*-value = 0.02). However, the recurrence and mortality rates were not affected by the blood group.^10^ In contrast to this study, Behtaj et al. reported that patients with non-O blood groups required more plasmapheresis to normalize platelet counts.^11^

In the study conducted by Yıldırım et al. in Turkey, 30 patients with acquired TTP were analyzed and blood group A was found to be a risk factor for immune TTP, whereas relapse and the requirement of plasmapheresis for remission were found to be higher in blood group O.^12^

In the current study, ABO and Rh blood groups were compared between the Control Group and the acquired TTP Group without giving any statistically significant difference. Furthermore, the O blood group, which was found to be less common in the TTP group than in the Control Group in previous studies, was found at a similar rate in this study (Control Group: 34.93 %, Acquired TTP Group: 32.3 %). Moreover, there was no statistically significant difference with the A blood group.

The AB blood group was found to be protective in terms of thrombosis and renal involvement, and it was found to decrease the creatinine level at the time of crises and improve platelet recovery, so it may be considered a good prognostic factor. However, it should be noted that fever was more common in the AB blood group. In addition, previous studies have shown that vWF antigen levels in the blood were high in patients with A and B antigens. It may be hypothesized that these vWF antigens compete with ultra-large vWF multimers at the receptor site and improve clinical symptoms. Alternatively, it could be hypothesized that an excess of vWF in the blood may act as a trigger for TTP and increase the incidence of the disease, which would explain the decreasing trend of TTP in individuals with blood group O as reported in the literature; further studies are needed.

The O blood group was associated with low platelet counts at the time of crises. In the study conducted by Hüssein et al., it was found that the O blood group required more sessions of plasmapheresis to reach remission than the B blood group. In this respect, although the frequency of TTP tends to decrease with the O blood group according to the literature, it can be said that the crises are more severe, so it may be an indicator of poor prognosis; this information needs to be confirmed with further studies.

The A blood group has been shown to increase the risk of relapse and thrombosis. In this respect, it may be an indicator of poor prognosis. However, the platelet count nadir at the time of crises was higher in patients with the A blood group, which also indicates a good prognosis.

The B blood group, in contrast to the A blood group, has been shown to decrease the risk of relapse and thrombosis. In this respect, it may be an indicator of a good prognosis. However, neurological involvement was more common, so it may also be an indicator of poor prognosis for neurological parameters.

The Rh antigen has been found to increase the involvement of fever. In this respect, it may be an indicator of poor prognosis.

The small number of TTP patients is one of the major limitations in this study as in other studies. The rarity of TTP limits the number of patients. While analyzing the relationship between blood groups and clinical parameters, the different treatments administered to the patients and different compatibility with the treatments and different follow-up periods also constitute limitations.

## Conclusion

This study demonstrated that the decreasing trend of blood group O in TTP patients is not observed in the Turkish population. It also showed that blood group A does not increase the risk of TTP, in contrast to a previous study conducted in Turkey. This research provides additional data by analyzing the relationship between blood groups and clinical parameters of TTP patients.

## Conflicts of interest

The authors have no conflict of interest.
